# A global-scale analysis of the sharing economy model – an AirBnB case study

**DOI:** 10.1140/epjds/s13688-022-00349-3

**Published:** 2022-06-29

**Authors:** Giovanni Quattrone, Natalia Kusek, Licia Capra

**Affiliations:** 1 Middlesex University, London, UK; 2 University College London, London, UK; 3 University of Turin, Turin, Italy

**Keywords:** Sharing economy, Airbnb, Market analysis, Linguistic analysis

## Abstract

The sharing economy model has changed the way in which people engage in a variety of activities, including travelling, trading, working, and lending/borrowing money. Several studies exist that aim to understand, quantify and model such phenomenon, but most such studies are geographically focused on countries in the Western World. Knowledge about the penetration and adoption of this novel market model in non-Western countries is much more limited, and almost completely lacking when it comes to emerging markets, where it was touted to bring the biggest benefits and be a game changer to uplift people economically. To close the gap, we chose Airbnb as an example of sharing economy model with worldwide market penetration, and performed a large-scale quantitative study of its penetration and adoption in seven cities in Asia, five cities in Latin America. We compared findings against seven cities in the Western World, and observed patterns to be similar across all locales, with two notable exceptions: the geographic penetration of such services, and the experience that guests travelling to such destinations shared in their reviews.

## Introduction

The sharing economy model [1], also known as collaborative consumption or peer-to-peer sharing, is an economic model that leverages the ability (and perhaps the preference) of individuals to rent / borrow goods and services rather than buying / owning them. It exists primarily as a *urban* phenomenon, since it is the density of cities that creates opportunities for underused goods and services to be available and within easy reach of matching demand. With no need for large, upfront financial investments, and only requiring access to information technology to take part, proponents of this economic model envisaged it would bring substantial benefits, including extra incomes for the users of such services, better resource allocation and utilization, and new economic activities for cities and municipalities. It was further envisaged that emerging countries would have been those benefiting the most from such economic model, as it could enable a more inclusive economic development, while also assisting with more transparent and fairer regulation and business formalisation [2]. However, from the outset, detractors worried that such economic model could cause more negative externalities than benefits, due to its predatory and exploitative nature, and with self-interest rather than sharing driving its functioning. They thus advocated for close scrutiny of services based on such economic model [3].

Fast forward 10+ years since its inception, the sharing economy model is now propelling a wide variety of services, from sharing a car in Uber, to renting a flat in Airbnb, from lending money in Kickstarter, to exchanging used clothes in Vinted. The exponential growth of such model has been accompanied by many public outcries: for example, Uber has been accused of unfair advantage over taxis [4]; Airbnb has been accused to be benefiting from lack of regulation and taxation compared to hotels [5]; and both platforms have been accused of being grounds for episodes of discrimination [6]. Several studies have thus ensued, that have taken a quantitative, data-driven approach, to understand, quantify, and model the penetration and adoption of some of the most popular sharing economy services, with the aim to objectively shed light onto many of these controversies (e.g., [7–9]). These studies and findings have offered governments, municipalities, as well the businesses themselves the ability to regulate their operation and market strategy in a more transparent and fairer way.

To date, most of these studies are however confined to services operating in cities in the Western World. The literature is significantly sparser, or completely missing, when it comes to non-Western cities. Take the case of Airbnb. This online accommodation platform is one of the most successful examples of sharing economy: according to statistics published in February 2022,^1^ there are at least 100,000 cities worldwide with Airbnb presence, over 5.6M active listings and over 1 billion guests having stayed at one of these. Yet, most of what we know of this service comes from studies of Airbnb conducted in either UK or US cities and we do not know whether Airbnb penetration and adoption is following similar patterns elsewhere. Studying sharing economy platforms such as Airbnb in other geographic contexts is important for different reasons: from an academic point of view, to generate knowledge about the relationship between urban geography and this economic model, at a time when technology and ease of movement have dramatically reduced barriers for cross-cultural interactions; from a practice point of view, to progressively inform the potential re-use and adaptation of policy and marketing strategies, as these services move out of their country of inception into lesser known realities.

The main aim of this paper is to reduce this knowledge gap, by performing a *global*-scale, quantitative analysis of Airbnb penetration and adoption. To this end, we collected 3.3 million Airbnb guest reviews about 220 thousand distinct listings, located in twelve non-Western cities that have been chosen to represent different continents, economies and cultures: seven in Asia (i.e., Bangkok, Beijing, Hong Kong, Shanghai, Singapore, Taipei and Tokyo) and five in Latin America (i.e., Belize, Buenos Aires, Mexico City, Rio de Janeiro, Santiago). We further collected 4.8 million Airbnb guest reviews about 205 thousand distinct listings across seven Western cities (i.e., Berlin, London, Madrid, Melbourne, New York, San Francisco, Vancouver), both to confirm previous findings and to have an up-to-date benchmark to compare against. For each city, we then studied:

- Airbnb *penetration*, measured both in terms of growth of offer (e.g., number of hosts sharing their property) and growth of demand (e.g., number of reviews that guests leave after their stays);
- Airbnb *adoption*, measured both in terms of satisfaction with the service (e.g., sentiment that guests express in their reviews) and appropriation (e.g., what aspects of the service guests discuss in their reviews).

We found similar patterns across all cities, with two notable differences:

- *geographic bias in service penetration*: in all cities analysed, we saw that offer and demand are not equally distributed across all neighbourhoods within a city, with more central and tourist areas having higher concentration than others. While this is to be expected to a certain extent, we observed a markedly lower geographic diversity in cities from Latin America. We hypothesise this is due to perceived lower levels of safety when venturing out of the most central and tourist districts, as well as higher difficulty moving around using public transports;
- *cultural and economic differentiation of experiences*: in all cities analysed, the sentiment expressed in guest reviews had a positively-skewed distribution, suggesting high satisfaction with the service overall. We also found that guests discuss different aspects of the service (e.g., location, facilities, communication with the host, hospitality) in equal measure in all cities, suggesting that travellers value the same aspects of the service wherever they travel. However, when looking at the sentiment expressed on a per aspect basis (rather than for the stay overall), we found markedly less positive sentiment for reviews left by guests visiting Asian cities. We speculate this might be explained both by *cultural* and *economic* differences: from a cultural point of view, extroversion is generally lower in Asian societies than in Western and Latin American ones, and this might manifest in the less positive sentiment associated to socially/interaction oriented topics such as communication with the host and hospitality; from an economic point of view, the housing unaffordability crisis that affects many Asian countries might manifest itself in the less positive sentiment associated to business oriented topics, such as value for money, facilities, and property.

The reminder of the paper is structured as follow: we first provide an overview of the many studies conducted in recent years of the sharing economy as a whole and Airbnb in particular (both outside the field of computing / data science, then specifically from a computing angle). We spell out the research questions that underpin this study, and detail the data we have collected from Airbnb in order to enable such investigation. We precisely define the various metrics we have computed to quantitatively explore each research question using the collected data, before delving into an extensive analysis of results. We conclude the paper with a discussion of our main findings and their implications, before we point out limitations and future directions of investigation.

## Related work

In the literature, terms such as digital economy, platform economy, and sharing economy have sometimes been used interchangeably. Although they do share some concepts, they also each have distinct characteristics and scope. We use the term *digital economy* [10] to refer to any economic activity that is facilitated by the use of computing technologies (and most importantly internet and the World Wide Web); in this sense, digital economy encompasses both platform and sharing economy, and has the widest scope. Within the digital economy, we use the term *platform economy* [11] to capture the more specific case where transactions are mediated by a platform (as opposed to, for example, emails). Examples of platform economy include DoorDash and Deliveroo. *Sharing economy* [1] adds a further nuance to platform economy, in that transactions do not only have the goal of matching supply and demand, but also comprise the idea of ‘sharing’ underused assets – from cars, as with BlaBlaCar, to houses and apartments, as in HomeExchange. Airbnb could be seen both as an instance of platform and of sharing economy, possibly due to the mixed nature of the accommodations it offers – spanning from entire properties that can be instantly booked (akin to platform economy services), to shared rooms in a flat (akin to sharing economy services). Indeed, some scholars consider Airbnb as an example of sharing economy, while others cast doubts on its ‘collaborative / sharing’ nature and prefer to classify it as an instance of platform economy instead [12]. In this paper, we consider Airbnb as an example of sharing economy, to capture its ‘asset’ sharing approach to renting.

While the debate of what precisely constitutes ‘sharing economy’ (SE) continues, there is no doubt this market model has been experiencing exponential growth, both in terms of the number of companies that adopt this model, and in terms of business value of its key players. It is thus no surprise that the number of academic studies on SE has also been growing exponentially, to the point that a number of scholars have started to conduct state-of-the art reviews of this body of work, both to understand the current stage of SE research and its future directions. The most recent and comprehensive of these review studies [13] analysed 219 academic articles published in the field, and developed a framework to help understand what we know so far of the phenomenon, as well as to signal open direction of investigations. The framework reveals that SE is a field being studied by many different disciplines interested in different aspects of the phenomenon: from economists, mostly interested in the business and reward models of the SE; to Law scholars, analysing the SE from a policy and regulation perspective; to sociologists, interrogating trust and motivation of the various stakeholders playing a role in it. The two business sectors currently dominating the SE landscape (that is, hospitality with Airbnb and transportation with Uber) also have been subject of extensive research from their respective disciplines.

The contribution that the computer science field has made to our understanding of the SE has however not been covered in the above. Even though sharing is an old practice, SE specifically is a rather recent phenomenon that has been propelled by information and communication technologies, and indeed scholars in the computing field have themselves been very actively studying the interplay between SE and computing. A survey [14] of 112 computing articles about the sharing economy has flagged two parallel research streams: one fundamentally technical, where algorithms are being proposed and evaluated to improve, for example, the matchmaking of the provider and consumer of a service (e.g., in the context of ride-sharing); and another one that is more socio-technically oriented instead, where the interplay between the computing platform that mediates SE interactions, and the individuals accessing the SE service, are being investigated. Our work falls within this latter stream.

A predominant research question within this stream has been motivation, trying to explain why peers choose to participate (or not) in the sharing economy. Studies pertaining over 40 different sharing economy services repeatedly found motivation to span widely, from more idealistic reasons (e.g., altruism, social connection) to more instrumental ones (e.g., value, the need to fulfil a need) [15, 16]. Different reasons were also found to be behind the choice not to engage in these platforms: from issues of safety and (dis)trust, to issues of independence and autonomy (or lack thereof) [17, 18]. Most of these studies used *a qualitative analysis approach* based on structured interviews and focus groups with a few selected participants; these methods afforded depth in the investigation of specific aspects or questions, to the expense of scale (both geographic and temporal). Further research is needed to understand whether motivation changes over time, and also to what extent motivation is linked to cultural and social norms, which vary from country to country. Geography has begun to be seen as an orthogonal lens of investigation and interpretation (for example, to understand different cultural, social and economic contexts), but so far it has mainly focused on the micro-scale (i.e., different neighbourhoods within a city, rather than different cities of the world). For example, geographic factors including population density and socio-economic differences across neighbourhoods were considered in a select few US Cities, and they were found to be strongly linked to differences in participation and success in major sharing economy platforms [19–21]; similar findings were also found for different neighbours in London, UK [22].

As large amounts of ready-available data capturing the interactions of participants in SE platforms are becoming available, *quantitative analysis approaches* have started to complement qualitative ones, so to analyse SE platforms at scale (both in terms of number of participants, geographic coverage, and over time). The vast majority of such quantitative studies have been about Airbnb, largely due to data availability. Indeed, studies of Airbnb have been proliferating so quickly to warren a dedicated systematic literature review solely on such platform [23]. For example, one stream of research has tried to quantify the impact that Airbnb presence has had on the city it penetrated, society or the tourism industry: specifically, a study of 13 cities in Italy [24] found the absolute majority of Airbnb listings to be concentrated in historical centres, a trend that has been increasing steadily over the years and that was linked to the ‘social desertification’ of Italian historical centres. A Texas-focused study [5] found causal evidence of the role that Airbnb played in the loss of hotel revenues. Other studies were however supportive of Airbnb in their findings: a UK-focused study [25] tried to quantify the impact of Airbnb penetration on local residents’ well-being, both from an economic, social and environmental perspective; surprisingly, it found a higher preponderance of positive effects (e.g., economic impact on the local community) than negative ones (e.g., fear of increased local crime). When zooming in London, UK, another study found strong evidence of the impact that Airbnb penetration had on increasing the value of real estate properties [26]. All these works offered evidence in the emerging debates between those in favour and those against SE services, ultimately aiming to help regulating such services. As SE services kept growing, other researchers have also looked at how SE platforms had been evolving over time, to understand what geographic, demographic and socio-economic factors are mostly associated with Airbnb adoption. Findings for several US cities of different size, wealth and population composition [21], and for the city of London, UK, [22] showed some strong similarities: areas of high Airbnb presence were usually those close to city centres and occupied by the ‘talented and creative’ classes; however, there were also important differences, with Airbnb penetration in London growing over time in more income deprived areas – a phenomenon that did not occur in any of the US cities analysed, thus signalling important geographic differences in the adoption of this platform. Another stream of research has focused on large-scale quantitative studies of satisfaction with the SE service, by means of statistical and linguistic analysis of ratings and reviews ([5, 27–32]). Results consistently found exceptionally high ratings and positively skewed sentiment in reviews; they also found ratings and reviews to be substantially higher in Airbnb as opposed to traditional economy platforms such as Booking.com and Tripadvisor, casting doubts on their actual value to Airbnb guests when deciding where to book a stay. While sentiment may not reveal much about an Airbnb listing, a more recent strand of investigation has taken advantage of advances in the field of natural language processing to conduct large-scale quantitative analysis of Airbnb reviews, this time to understand what participants in this hospitality service care the most about ([33–39]). Business-oriented aspects such as location, facilities/amenities, and communication with the hosts were consistently found to be particularly important in all studies, while price was rarely discussed in reviews. Interestingly, the more social-oriented aspects of the Airbnb service (i.e., interactions between hosts and guests) steadily lost importance over time [39], suggesting that Airbnb has been increasingly evolving into a ‘platform economy’ as opposed to a ‘sharing economy’ one. By complementing text mining techniques on the few negative reviews present in the platform with focused interviews, a recent study [40] looked into identifying causes of Airbnb service failure (e.g., poor guest-host interaction, quality of the room not as described); interestingly, service recovery strategies were found to be very similar to those expected for hotels (e.g., compensation, apology).

A common limitation to all quantitative studies mentioned above is their geographic focus: there is a strong geographic bias towards North-American cities, some studies in Australasia and Europe (mostly UK cities), but the literature becomes suddenly very sparse as one looks further afield. Indeed computing scholars have flagged the need to study the SE phenomenon in non-Western markets as one of the most urgent directions of open SE research [14, 20]. Even Airbnb, the most widely studied SE platform, is in need of cross-cultural investigations, as flagged by a recent systematic review if this platform [23]. This is because the SE is fundamentally a *urban* phenomenon, and local geographic factors ranging from urban structure to population density patterns to social structure might significantly impact many of the phenomena of interest in the sharing economy literature (including user motivation, adoption and optimization). Indeed there is a possibility that some of the findings in the current literature corpus would change if studies were repeated in new geographic contexts.

With the present study we aim to make a step towards closing this knowledge gap, so to better understand the relationship between local geography and Airbnb adoption across the globe.

## Research questions

In this work, we perform a global-scale *urban* study of Airbnb penetration and adoption. We focus on cities, as opposed to suburban or rural areas, since this is where the vast majority of Airbnb activity takes place. Indeed, several studies about the spatial penetration of Airbnb confirm that hosts and guests cluster around city centres and the most tourist areas (e.g., [21, 41, 42]). We are then concerned with two main research questions:

*RQ1:* *what does the urban penetration of Airbnb look like in different cities around the world?* As mentioned in the Related Work section, previous studies of Airbnb in Western World cities highlighted that neighbourhoods in the city centre and the more tourist areas benefit the most from this economic model, getting the lion share of guests, while more peripheral and less tourist areas benefit significantly less from this model. Furthermore, a small number of hosts seem to amass a disproportionately high number of reviews (i.e., guests stays) compared to others, thus casting doubts on the claim from supporters of such economic model that it enables a fairer distribution of wealth in society. Since past studies have mostly focused on cities located in the Western World, we presently lack knowledge of what the urban penetration of Airbnb looks like in other geographic and socio-economic contexts. With this study, we aim to start closing this knowledge gap, and to gather evidence of who benefits from this economic model in different locales, which in turn would help both sharing economy companies like Airbnb to understand context-specific opportunities for growth and marketing, and local authorities to inform policies for regulation and taxation.
*RQ2:* *how is Airbnb being adopted in different cities globally?* Studies of Airbnb in Western cities have revealed that the hospitality service offered is mostly enjoyed by hosts and guests alike, given the extremely high star ratings that both ends of the service receive, and the highly positive sentiment expressed in the reviews they publicly exchange. Furthermore, the hospitality service offered is very much business oriented, with guests particularly looking for convenient location, ease of booking and communication (as opposed to, for example, a more socially/interaction oriented experience between hosts and guests). What we do not know is whether Airbnb is being adopted in the same way in cities with different cultures and different socio-economic contexts. Once again, the aim of this study is to reduce this knowledge gap, and to gather evidence about the perceived quality of such services in other markets. Findings could inform changes within the sharing economy company to make its operations more successful in different locals; they may also offer evidence to governments based on which to decide whether to welcome and support these SE businesses or not.

## Airbnb data

To conduct a global scale study of Airbnb, we started by selecting twelve cities in non-Western countries across the globe: Bangkok (Thailand), Beijing (China), Belize (Belize), Buenos Aires (Argentina), Hong Kong (China), Mexico City (Mexico), Rio de Janeiro (Brazil), Santiago (Chile), Shanghai (China), Singapore (Singapore), Taipei (Taiwan) and Tokyo (Japan). These cities have been chosen to cover different geographical regions (e.g., seven cities in Asia and five cities in Latin America), different economies (e.g., wealthy cities like Tokyo as well as small, developing economies like Belize), and different cultures (e.g., collectivist and long-term oriented cultures where people want to feel more anonymous, be part of the larger community and unlikely to freely offer complaints – such as Beijing, as well as short-term oriented collectivist cultures like Rio de Janeiro, where people communicate in a more expressive manner). Airbnb data for each of these cities was scraped directly from the Airbnb website in June 2021. Since the use of the platform was suddenly brought to a near halt once the COVID-19 pandemic stroke, due to international travel bans and severe restrictions on people mobility, we chose to exclude from the present study any Airbnb stay that occurred from January 2020 onwards (that is, once the pandemic status was announced). Separate studies will be required to understand both short (temporary) and long term changes in Airbnb behavioural patterns deserve post pandemic. For the present study and the twelve cities listed above, we collected 3.3M guests reviews about 220K unique listings.

At the same time, we also collected Airbnb data about seven cities in the Western World, to use as baseline comparison: Berlin (Germany), London (United Kingdom), Madrid (Spain), Melbourne (Australia), New York (United States), San Francisco (United State), Vancouver (Canada). For these, we collected a total of 4.8M guests reviews and 205K unique listings.

All collected data was subjected to a cleansing process. First, we removed reviews automatically generated by the system in the case of cancellation (around 0.8% in non-Western cities and 1.3% in Western cities), as well as reviews without comments (around 0.2% for both Western and non-Western cities).

In exploring *RQ2*, our method of analysis (described in detail in the next section) is based on linguistic analysis of reviews. For this part of the study, we went a step further with our cleaning process and proceeded by detecting the language in which reviews were written, and removing those that were not in English. In so doing, we were left with 82% of reviews in the seven Western cities, and 39% in the remaining twelve cities. Indeed we found that, in most non-Western cities, the most frequent language used in reviews is the country’s official language (with the exception of Belize and Singapore, where English is by far the most common language used in reviews instead). It is a limitation of this study to only focus on English reviews for RQ2 – in so doing, we miss a large fraction of the Airbnb user base in non-Western cities, and future studies should remove this limitation and advance knowledge by studying reviews in the local language too. However, by focusing on English reviews only, what we can do is to study the adoption of Airbnb across the world from the point of view of the same ‘type’ of traveller (i.e., those who write their reviews in English).

Table 1 provides a summary of the data collected for each of the twelve plus seven cities under study.

**Table 1:** Listings and reviews in Asian, Latin American, and Western cities

City	# Listings	# Reviews	% English Reviews
*Asia*			
Bangkok	17,040	249,681	66%
Beijing	36,864	258,218	10%
Hong Kong	11,449	203,875	51%
Shanghai	29,165	347,268	10%
Singapore	3672	43,239	83%
Taipei	8409	283,690	41%
Tokyo	15,009	398,181	63%
*Latin America*			
Belize	2949	45,039	95%
Buenos Aires	23,828	384,976	35%
Mexico City	21,662	527,451	44%
Rio de Janeiro	35,793	348,895	29%
Santiago	16,118	211,162	25%
*West*			
Berlin	17,290	412,678	66%
London	85,207	1,472,733	87%
Madrid	17,831	643,882	49%
Melbourne	23,862	614,589	95%
New York	49,530	1,182,158	88%
San Francisco	6413	285,275	93%
Vancouver	5806	203,284	93%

## Metrics

### Research question #1 - penetration

We investigate Airbnb penetration from two perspectives:

*Geographic penetration*: we first look at what *areas* within a city benefit the most from Airbnb. To do so, we compute three complementary metrics:

- *offer density* - i.e., number of unique Airbnb properties per area, normalised by area size. This metric allows us to inspect the spatial distribution of Airbnb properties in a city, and to observe whether offer is evenly spread or whether certain areas have a bigger presence on this hospitality platform;
- *demand density* - i.e., number of guest reviews per area, normalised by area size. Offer density tells us where properties are made available (i.e., what areas are *potentially* gaining from the Airbnb market model), but it does not tell us whether these properties are actually being used. With demand density, we inspect the spatial distribution of actual stays. Note that we do not have booking data, but past studies suggest that more than 70% of stays result in guests leaving reviews, so we consider reviews to be a good proxy for demand [43];
- *area inequality* - i.e., the degree of variation of Airbnb demand within a city, measured as the Gini coefficient of the number of guest reviews among the different areas within a city. The Gini coefficient is a measure of statistical dispersion and in this context it aims to capture the wealth inequality that different urban areas experience from Airbnb guest stays.

When computing the above metrics, we use as our spatial unit of analysis the Airbnb definition of neighbourhoods.^2^ The boundaries of such neighbourhoods have been determined by Airbnb based on research with cartographers, locals and city experts, and are maintained accurate and up-to-date also based on feedback from Airbnb users.

*Host penetration*: we then move our attention from areas to *hosts*, and investigate to what extent Airbnb demand is evenly distributed among them. To do so, we compute a *host inequality* metric as the Gini coefficient of the number of guest reviews among the different hosts within a city, thus obtaining a complementary view with respect to the area inequality metric defined above. Note that one host could manage more than one listing in Airbnb; our analysis is performed at host level (as opposed to property level), thus accounting for multiple property ownership.

### Research question #2 - adoption

We study how the Airbnb hospitality service is being perceived and adopted in different cities around the world based on what transpires from the reviews that guests leave after a stay. For this research question, we consider the whole city as our unit of analysis. We then focus on guest reviews written in English and pursue two streams of investigation:

*Sentiment analysis*: we first investigate how the overall *guest satisfaction* with the Airbnb hospitality service varies between different cities. To do so, we use VADER (Valence Aware Dictionary for Sentiment Reasoning), an NLP algorithm that is capable of capturing both polarity (positive / negative / neutral) and intensity (strength in $[0,1]$) of emotions in text [44]. VADER has been shown to work particularly well on short text, so we first break down guest reviews into sentences, then run the VADER sentiment analyser on each of these, and obtain a numeric score for each of the three categories (i.e., positive / negative / neutral). VADER also returns a compound score for each sentence, that is computed by normalising the previous ones. In the Results section, we will report the mean compound sentiment score at city level.

*Topic analysis*: we then delve deeper, and investigate what travellers to different cities care about the Airbnb hospitality service, and how satisfied they are with each aspect of the service received.

To find out what aspects of the Airbnb service guests care about, we used the Gibbs Sampling Dirichlet Mixture Model (GSDMM) topic modelling algorithm [45] to find, in an unsupervised way, what topics are most frequently discussed in guest reviews. We chose GSDMM as opposed to the more popular Latent Dirichlet Allocation (LDA) topic detection algorithm, since the latter is known to have poor performance on short texts (something that we also verified when we first used this on Airbnb reviews). We run the GSDMM algorithm on the whole corpus of (English) guest reviews, and derived a common set of topics we can use to analyse and compare cities across the globe. The optimal number of topics has been determined according to a heuristic approach based on the perplexity score [46]; in this context, it was found to be equal to 10. Three independent human annotators inspected the 20 most common words belonging to each topic, as well as the sentences that had the highest probability (according to the output of GSDMM) to belong to each topic, and then assigned meaningful, human-interpretable names to each topic. The GSDMM model was then used to assign a probability $p(s,t_{i})$ to each sentence *s* in the corpus to belong to each of the 10 detected topics $t_{i} \in [1,10]$. We then defined the overall topic importance *ti* of each of the 10 topics in each of the cities under study, as the mean value of $t_{i}$:

$$ ti(t_{i}, city) = \mbox{mean}\bigl( p(s,t_{i}) \bigr), \quad s \in city $$

Finally, to find out how satisfied guests were with each aspect of the service, we grouped all sentences associated with topic $t_{i}$, used VADER to compute the sentiment associated to it, then returned the mean sentiment (per topic, per city).

Before conducting the linguistic analysis described above (on both sentiment and topic), a number of data cleansing steps took place: in particular, non-ASCII characters and numbers were removed, and contractions were expanded; punctuation was used to break down reviews into sentences, and sentences were lemmatised then analysed for their length. Finally, sentences that were either too short (less than 8 words) or too long (more than 97.5th percentile — more than 175 words) were removed: too short reviews do not hold enough information to extract sentiment or topic and are often a source of noise; on the other hand, too long reviews may disproportionally influence the outcome of the NLP algorithms used to compute sentiment and topic. In total, about 10% of sentences were removed from reviews.

## Results

### RQ1 - penetration

#### Geographic penetration

**Figure 1:**
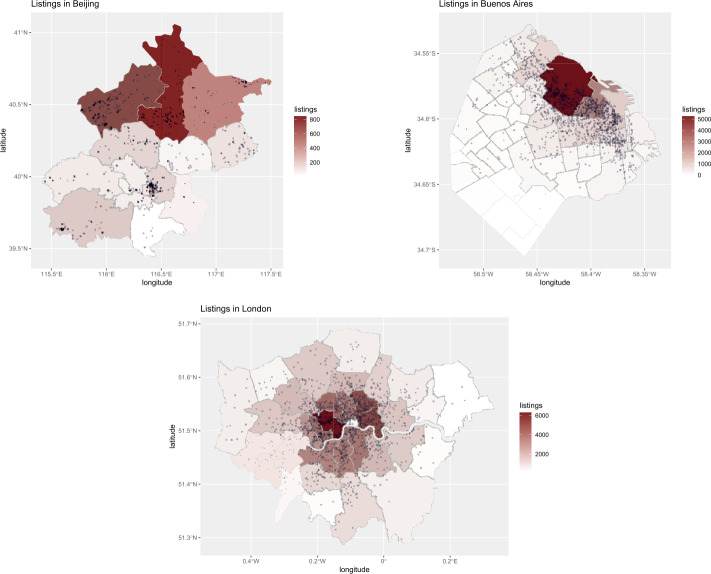
Offer (host) density in Beijing, Buenos Aires and London

**Figure 2:**
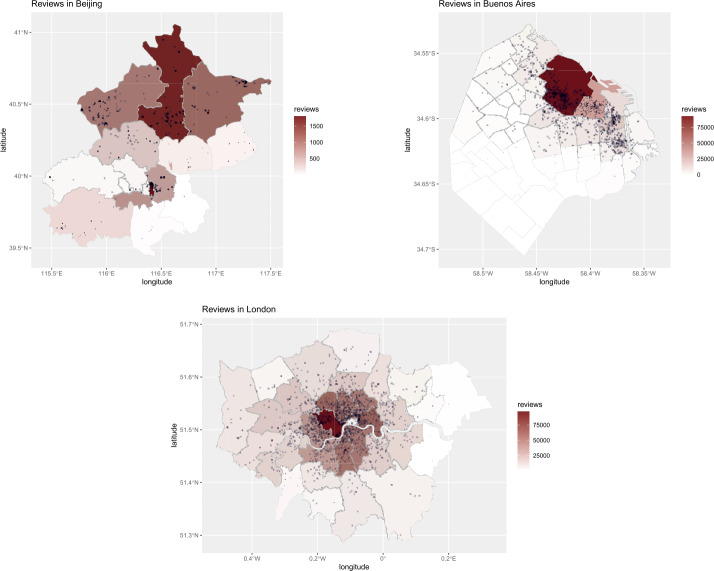
Demand (guest) density in Beijing, Buenos Aires and London

Previous studies of Airbnb penetration in Western cities had found that offer and demand are not equally distributed across all neighbourhoods within a city, with more central / tourist areas having significantly higher concentration than others. We now have evidence that geographic bias is present in Asian and Latin American cities too. For example, Figs. 1 and 2 visually illustrate penetration of hosts and guests respectively, for three representative cities: Beijing in Asia, Buenos Aires in Latin America, and London in the West.^3^ We note that, in Beijing, Airbnb hosts and guests are all heavily clustered around the Dongcheng district (where one of most famous tourist destinations, that is, The Forbidden City, is located); in Buenos Aires, they are clustered around the Monserrat neighbourhood (where Plaza de Mayo is located); in London most hosts and guests are also clustered in the city center. However, we can observe that such geographic bias is markedly stronger in Latin American cities (here represented by Buenos Aires), with almost no host and no guest presence at all outside the most tourist district.

**Figure 3:**
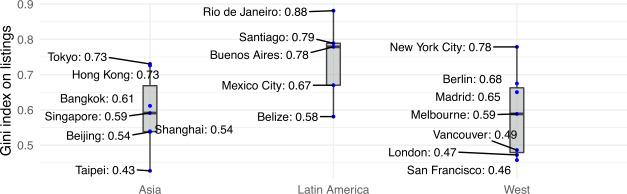
Host inequality measured as Gini coefficient of the number of listings per area

**Figure 4:**
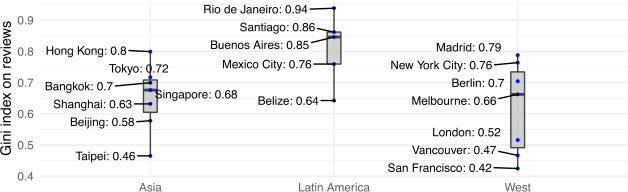
Guest inequality measured as Gini coefficient of the number of reviews per area

To quantitatively measure inequality of hosts’ and guests’ clustering within a city, we have computed the Gini index [47] for both offer (i.e., hosts’ listings) and demand (i.e., guests’ reviews) for all cities under study. Results are reported in Fig. 3 and Fig. 4 respectively.

As shown, offer inequality is higher for Latin American cities (Wilcoxon rank test *p*-value <0.01), where the median Gini coefficient is 0.78 (from a minimum of 0.58 for Belize to a maximum of 0.88 for Rio de Janeiro). Elsewhere, the median host inequality is 0.59 (for both Asian and Western cities), with a peak of 0.78 for New York City.

To investigate whether there exist a few neighbourhoods who amass the largest share of Airbnb guests, Fig. 4 reports the Gini coefficient for demand (i.e., guest reviews) for all cities under study. Once again, cities in Latin America exhibit greater inequality than Asian and Western cities (Wilcoxon rank test *p*-value <0.01). Notably, Rio de Janeiro in Latin America leads the ranking as the most unequal city (Gini coefficient equal to 0.88); whereas San Francisco (a Western city) and Taipei (an Asian city) are the most equal ones (Gini coefficients 0.46 and 0.43, respectively).

**Figure 5:**
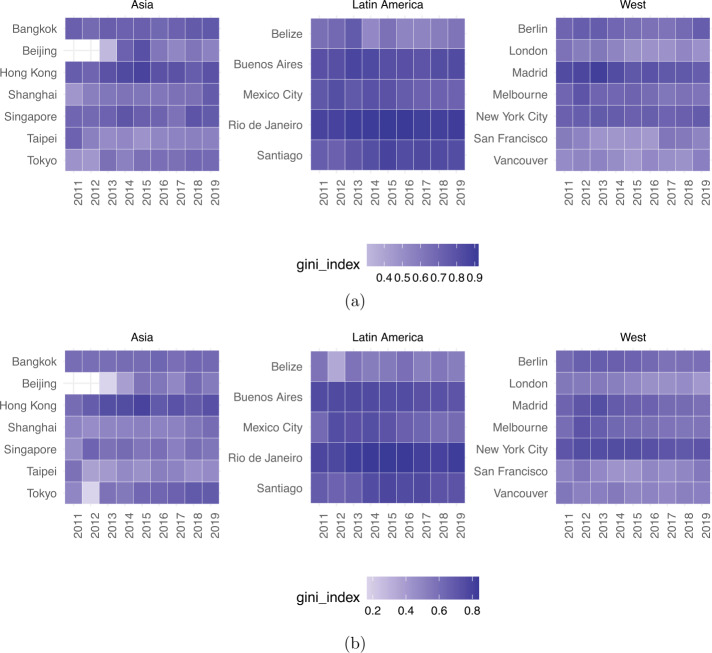
Geographic inequality measured as Gini coefficient of the number of reviews (plot a) and number of listings (plot b) per city and per year

We also measured the Gini coefficient on a per year basis (from 2011 to 2019 – Fig. 5), both for hosts’ listings and for guests’ reviews, to check whether inequality was on the rise or decline: in both cases, after the first few years of instability in a few Asian cities (the early stage of ‘platform adoption’), inequality stabilises and has remained flat over time (up to 8% of Gini coefficient variation from 2013 to 2019). Note that Fig. 5 (as all as all subsequent figures) only show results when we have at least 1K reviews per city per year; this is because our sensitivity analysis (see Sensitivity section later on) found 1K to be the minimum number of reviews needed to have robust and statistically significant results.

#### Host penetration

**Figure 6:**
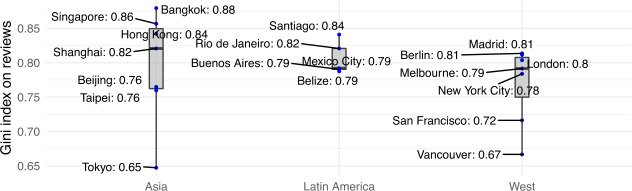
Host inequality measured as Gini coefficient of the number of listings per host

We also analysed inequality at host level, to investigate whether there exist a few hosts who amass the largest share of benefits from Airbnb guests. We did so by measuring the Gini coefficient of the number of reviews received *per host*, as opposed to per city area. Results for each city, aggregated over all years under study, are summarised in Fig. 6: in this case, inequality is very high (median value above 0.79) in all geographic regions, with no significant difference between Asia, Latin America and the West. Noticeable exceptions are Vancouver and Tokyo, with slightly lower Gini coefficients (just above 0.65).

**Figure 7:**
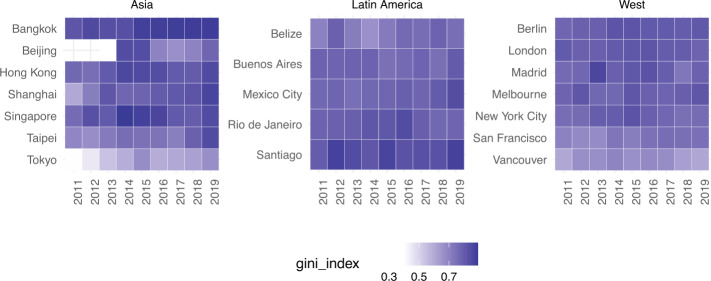
Host inequality measured as Gini coefficient of the number of listings per host per year

As previously done for area inequality, we also measured this Gini coefficient on a per year basis (Fig. 7); as before, apart from the first couple of years of platform instability, the levels of inequality have remained flat at very high levels everywhere (up to 5% of Gini coefficient variation from 2013 to 2019).

### RQ2 - adoption

**Figure 8:**
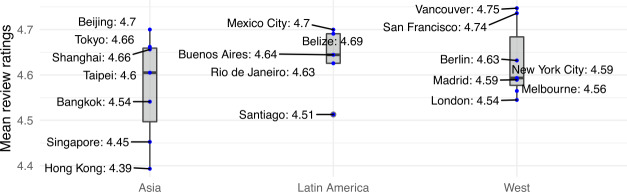
Average reviews’ ratings

*Sentiment Analysis.* It has previously been reported that Airbnb stays in Western cities are very highly rated, with an average score of 4.7 (in a scale $[1,5]$), as opposed to an average of 3.9 as reported on other platforms such as TripAdvisor [48]. As Fig. 8 illustrates, guests’ numeric ratings are very high for stays in Asian and Latin American cities too (the same holds when disaggregating reviews by city and by year).

**Figure 9:**
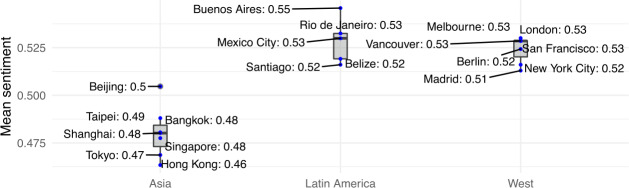
Mean value of Sentiment Analysis

**Figure 10:**
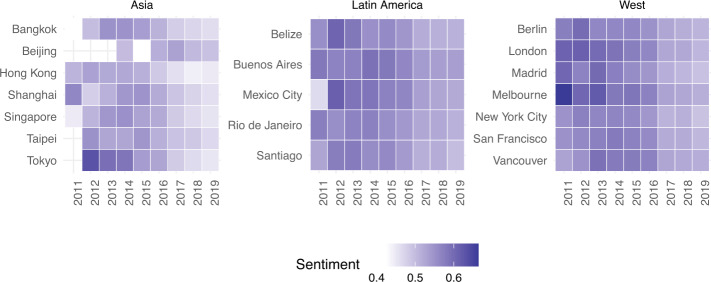
Evolution of Sentiment Analysis over time

To delve deeper into actual guest satisfaction, we then conducted a sentiment analysis of the reviews they left after their Airbnb stays. Figure 9 illustrates mean review sentiment score as determined by the VADER sentiment analyser, as described in the Metrics section. A much more nuanced picture now emerges: while Western and Latin American cities have a mean sentiment value above 0.525, Asian cities have a significantly lower mean sentiment (Wilcoxon rank test *p*-value <0.01), hovering just above 0.475. These values remain roughly the same even when disaggregating reviews on a per year basis (Fig. 10), suggesting that sentiment has only slightly decreased over time in all cities under analysis.

*Topic Analysis.* To delve deeper into what Airbnb guests care about, we performed a preliminary topic detection step, considering all English-written reviews together for all cities under study. We detected 10 distinct topics; Table 2 illustrated the lists of topics we obtained and the 8 most relevant words associated with each of them. Note that each topic in Table 2 is shown through a human-interpretable ‘topic name’ (hereafter simply referred to as topic).

**Table 2:** 10 detected topics and their 8 most relevant words

Topic	Top 8 words
Communication with host	check, host, communication, respond, question, arrival, answer, response
Description accuracy	place, space, apartment, picture, room, describe, photo, look
Experience	would, stay, place, recommend, enjoy, time, thank, visit
Facilities	provide, breakfast, towel, food, kitchen, coffee, tea, touch
Hospitality	host, stay, home, feel, enjoy, family, hospitality, welcome
Host advice	host, tip, city, recommendation, suggestion, help, area, information
Location	location, minute, location, walk, station, restaurant, shop, neighbourhood
Property	room, bed, shower, space, bathroom, kitchen, living, water
Surroundings	building, night, parking, city, street, noise, window, sleep
Value for money	value, space, room, locate, explore, money, price, stay

Six of these topics cover business-oriented, professional aspects of the hospitality service (these being ‘Description accuracy’, ‘Property’, ‘Value for money’, ‘Location’, ‘Surroundings’, ‘Facilities’). Three of these topics cover social (interaction) aspect of the hospitality service (these being ‘Communication with host’, ‘Hospitality’, and ‘Host advice’). The tenth and final topic is neither about the business-side of the service nor about the interaction (social) side of the service, but it is about the individual ‘Experience’ of the guest with their stay (i.e., the part of the review where guests discuss whether they *enjoy*ed their *stay* and whether they would *recommend* it).

**Figure 11:**
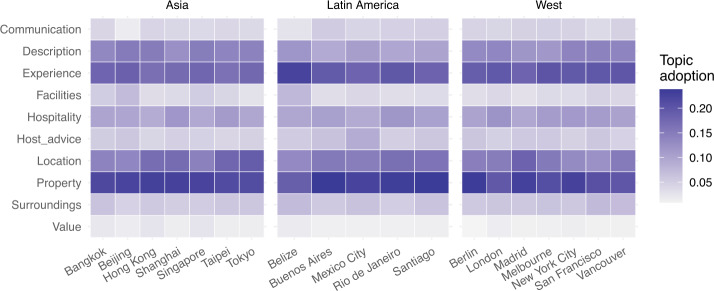
Topic importance

When we look at what topics are discussed most frequently, we find that there is no significant difference in terms of relative importance of the topics discussed in guests reviews among the cities under study. In all cities the topics ‘Experience’ and ‘Property’ strongly dominate (Fig. 11). After these, two business-related topics follow: ‘Description accuracy’ and ‘Location’. We note that the least discussed topics are the ones about ‘Value for money’, ‘Communication with host’ and ‘Facilities’ (where, for example, the *kitchen* is mentioned; whether *breakfast*, *coffee*, *water*, and *towels* are provided; and so on). The latter result is possibly because the Airbnb platform already provides guests with detailed information about what facilities they should expect to find in the property selected, so when such facilities are discussed in reviews, it is more often to reflect on whether the description was accurate or not (as signalled by the more popular topic ‘Description accuracy’).

**Figure 12:**
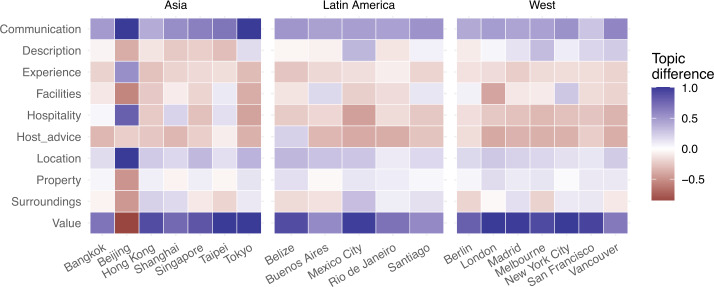
Percentage change of topic importance from 2015 to 2019

We also analysed *variation* of topic importance between 2011–2015 aggregated, and 2016–2019 aggregated, to understand whether guests are changing what they care about in their stays (from what can be inferred from the reviews they write). As Fig. 12 illustrates, two topics have clearly gained importance in this time span: ‘Communication with the host’ and ‘Value for money’. Variation of importance for the other topics is much smaller instead; in fact, some social/interacting aspects of the service, such as ‘guest advice’ and ‘hospitality’, only slightly declined over time. One city stands out as an outlier: Beijing, for which we note most topics have indeed changed relative importance, with significant gain for ‘Experience’, ‘Hospitality’ and ‘Location’, and loss for ‘Value’, ‘Property’, ‘Surroundings’ and ‘Facilities’. These topic changes might be attributed to the fact that Beijing was in the early stages of technology adoption during 2011–2015, and thus fluctuations might be expected.

**Figure 13:**
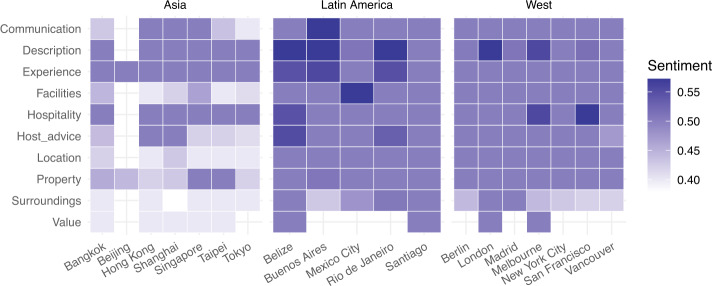
Mean sentiment aggregated by topic

As a final step in our investigation of similarities and differences of Airbnb adoption around the world, we then analysed the *sentiment associated with each topic* in each city under study. Figure 13 illustrates the mean sentiment we measured by topic and by city.

This time a significant difference emerges: in Asian cities (with the exception of Beijing having not enough reviews to draw any conclusion), the sentiment associated with any of the 10 topics is lower than elsewhere in the world, reflecting also what we discovered when analysing sentiment of the review overall. As we delve deeper, we can see topics ‘Description accuracy’, ‘Experience’, and ‘Hospitality’ have the highest sentiment, while ‘Location’, ‘Surrounding’, and ‘Value’ have the lowest. We propose a possible interpretation of this finding in the Discussion section.

### Sensitivity analysis

In order to gain confidence in the robustness and validity of our results, the following sensitivity analysis has been conducted:

- *Removing too short (i.e., less than 8 words) and too long (i.e., more than 175 words) reviews*. We have considered different values for these thresholds, by randomly increasing or decreasing them up to 50% of their original value. All results we got were very consistent, showing no significant change of detected *sentiment* aggregated by city and year (stable values having a maximum variation of roughly 1%). Similarly, no significant change was found for *topic* adoption aggregated by city and year (stable values having a maximum variation of roughly 2% in very few occasions).
- *Removing non-English reviews*. In many non-English speaking cities, a large portion of reviews has been removed from our analysis. Notwithstanding that our results reflect only English speaking guests and that our conclusions cannot be extended to non-English speaking ones, we questioned whether our filtering could have removed statistical significance from our results. As an example, in Beijing only 10% of reviews are written in English, leaving us with only 25K reviews to analyse (down from the original pool of roughly 250K reviews). In particular, we investigated what is the minimum number of English reviews per city per year that is needed to get statistically significant results.
  To answer this question, we considered the cities having the highest number of English reviews: London, New York, Melbourne, Madrid, Berlin. We grouped reviews by city and year, and for each group we sampled data by considering different sample sizes: 500, 750, 1K, 2K and 5K reviews per sample. For each sample size, we then computed the sentiment and topic adoption measures ten times, with each iteration operating on a different random sample. Finally, we measured the consistency of our results in terms of the *coefficient of variation* *CV* (i.e., the ratio of the standard deviation to the mean), thus measuring the variability of results with respect to the population mean. We found that, when a sample contains fewer than 1K reviews, the coefficient of variation *CV* associated with sentiment and topic adoption is generally greater than 0.1, indicating that the obtained results are starting to be less reliable. For this reason, in this paper we only reported results per city per year when we had at least 1K reviews to analyse (i.e., for which we got $CV <0.1$ and therefore we consider our results to be reliable).

## Discussion

Our investigation of *Airbnb penetration* in cities around the world has revealed significant evidence of *geographic bias* in both Western and non-Western cities; however, such bias is markedly stronger in non-Western cities (Latin America in particular), with almost no Airbnb presence outside the single main tourist district. We may speculate this is linked to lower levels of safety (either real or perceived) outside such areas, as well as less developed public transport infrastructures, that make tourists less likely to venture (even just slightly) further away for their stays, as they do in Western cities such as London and Vancouver instead. In order for sharing economy services such as Airbnb to penetrate beyond city centres, and for local neighbouring areas to benefit from such economic model, government investments in a safe, efficient, capillary public transport infrastructure need to happen first [49, 50]. Major events may be strategically used to achieve such urban development objectives; in the past, these events were known to generate positive benefits that were inequitably distributed, but there have also been examples whereby careful planning of initiatives and developments to accompany the major event have lead to the outcomes being reciprocal benefits for both central and more peripheral stakeholders [51, 52]. Local sharing-economy stakeholders and governments should partner and work together to develop long-term investment plans in peripheral urban areas so that both sides can benefit from them (in the form of a more equitable distribution of wealth across a city).

Our investigation of *Airbnb penetration* in cities has further revealed substantial *host inequality* in all cities analysed – that is, there exist a few hosts that amass a disproportionally high share of reviews, suggesting the economic benefits of Airbnb are in the hands of a few, as opposed to be evenly distributed among many, as the sharing economy model would aim to achieve. This is despite hosts being equally centrally located (as the majority are at the moment), and reviews being equally positive across all stays. This may be due to Airbnb use of ratings and reviews as the platform trust-building mechanism: that is, the hosts that appear most trustworthy in the eyes of potential guests are those who have gathered the highest number of (positive) ratings and reviews. This invariably leads to a rich-get-richer phenomenon in all cities analysed, where a small number of popular hosts (possibly those who joined the platform first) grow their popularity exponentially, while new hosts struggle to attract any stays. This loop can however be broken with appropriate policies and incentive schemes aimed at a more democratic and equitable distribution of wealth among hosts: for example, a ‘new host referral scheme’ could be deployed to encourage popular hosts to introduce new hosts they know to the platform, thus acting as guarantor for them for some economic benefit in return; a ‘first stay discount scheme’ could be used to attract guests to stay with hosts who are new to the platform, for a discounted rate; furthermore, the platform could use an internal recommender system that dynamically ranks and displays properties to searching guests so to value host equity too.

In terms of *Airbnb adoption*, our analysis has revealed overall very high satisfaction with this hospitality service, as indicated by highly positive-skewed distributions of both ratings and sentiment expressed in guests’ reviews in all cities under study. Our topic analysis also suggests that travellers care about the same aspects of the service to the same extent, no matter what city they travel to. This is because we observed the same frequency distribution of topics in all cities under study: guests discuss first and foremost their own ‘Experience’ overall; they then delve into more business-oriented aspects of the service, such as ‘Property’, ‘Location’, and ‘Description accuracy’. Social/interaction aspects of the service, such as ‘Hospitality’ and ‘Host advice’ are being discussed to a lesser extent instead, and this is the same everywhere. Recent findings [39] suggesting that the Airbnb hospitality service is a business-oriented enterprise (akin to hotels and rental accommodation services), as opposed to a socially-oriented one (unlike their early days, when the business seemed to be all about sharing spaces and experiences) appears to be confirmed beyond Western cities. This also tallies with various reports noting how Airbnb has been aggressively trying to appeal to business travellers and families, whose needs are undoubtedly different from the younger guests who used to sleep on sofas or spare rooms when the start-up began [53].

Despite this high degree of homogeneity of *Airbnb adoption* across the world, *local differences* do emerge as one delves deeper and starts looking at the sentiment expressed in guest reviews. In particular, we found markedly less positive sentiment for reviews left by guests visiting Asian cities than anywhere else in the world, both in terms of overall sentiment and in terms of sentiment associated to both business and social aspects of the service. We hypothesise this might be explained by economic and cultural differences: from an *economic point of view*, the housing unaffordability crisis that has been affecting many Asian countries [54] might manifest itself in the less positive sentiment associated to business-oriented topics, such as ‘Value for money’, ‘Property’, and ‘Facilities’. From a *cultural point of view*, extroversion is generally lower in Asian societies than in Western and Latin American ones, and this might manifest in the less positive sentiment associated to socially-oriented topics such as ‘Communication with host’, ‘Hospitality’, and ‘Host advice’. Airbnb could help its guests set their expectations right before travelling to a foreign city: at the moment, the platform offers “city guides” to help explore the city once there, but these guides could be expanded to offer travellers a better understanding of the local socio-economic context, as well as local culture, before a booking is even made.

## Limitations & future work

The main limitation of this work concerns the linguistic analysis we have conducted. In our investigation on Research Question #2 (Airbnb adoption), sentiment and topic analysis have been conducted on English-written reviews only. In the non-Western cities studied, these represent only 39% of the total reviews left by guests (with ‘local’ languages such as Spanish, Portuguese and Mandarin being the dominant languages instead). In so doing, we have been able to study similarities and differences of Airbnb adoption across the world, but only from the point of view of English-writing guests. In a sense, we have used the English language as a control factor for the groups of guests whose reviews we have been following around the world.

What this study has not done is the complementary investigation: that is, study similarities and differences of Airbnb adoption between different groups of guests travelling to the same city. To do this, one would select a city (to act as control), and then study all reviews left, segmented by the language used in the review. By repeating this complementary study for several cities around the world, one would then be able to provide a more complete picture of Airbnb adoption. We leave this study for future work.

Although this study has expanded our knowledge and understanding of Airbnb penetration and adoption beyond Western countries and into Asia and Latin America, there is still a substantial knowledge gap when it comes to developing countries. Some initial research work has occurred in Namibia and Kenya (e.g., [55–57]), suggesting that the sharing economy is playing a rather different role in developing countries than developed ones; this is calling for significant more attention and research in the immediate future, not just to understand what local geographic factors are key determinants of the performance of SE platforms in these contexts, but critically to also assess whether such factors might also cause biases in the demographics that benefit from the SE.

Last but not least, these studies should be repeated regularly over time to detect, understand and adapt to changes, as they might be driven by a variety of factors: from changes in legislation about this type of services, to crisis like the most recent COVID-19 pandemic. Although actual findings may change, the methodology proposed and used in this paper remains, as it enables this type of investigations to easily scale up and be repeated at varying units of spatio-temporal granularity.

## Figures and Tables

**Figure 14:**
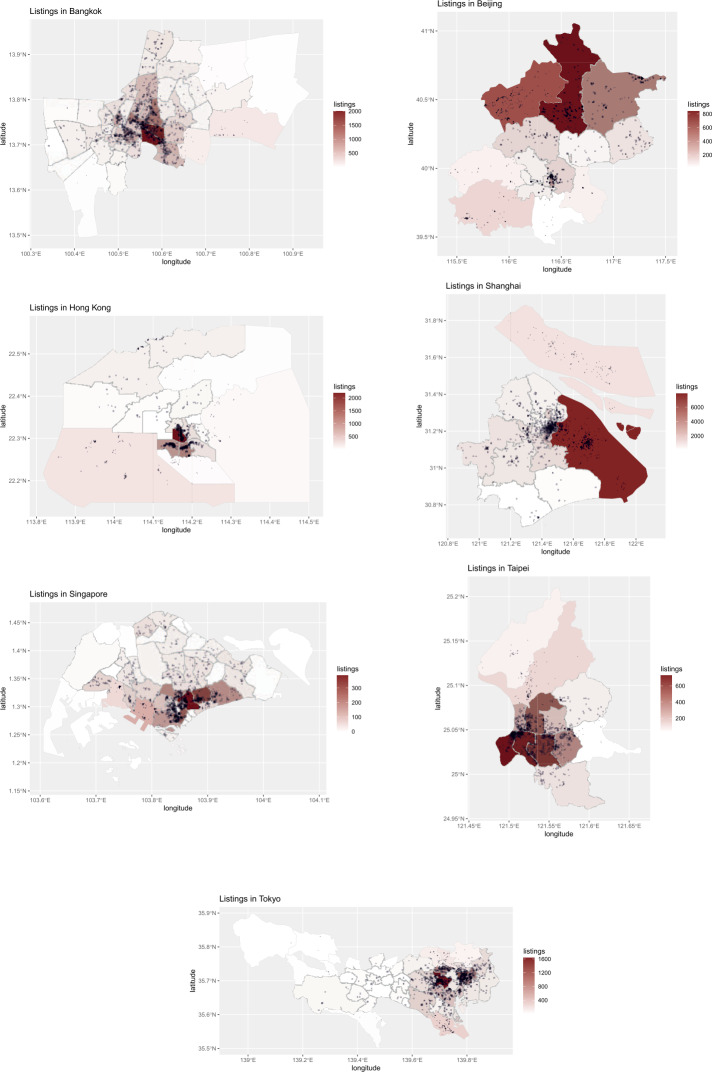
Offer (host) density in the seven Asian cities analysed

**Figure 15:**
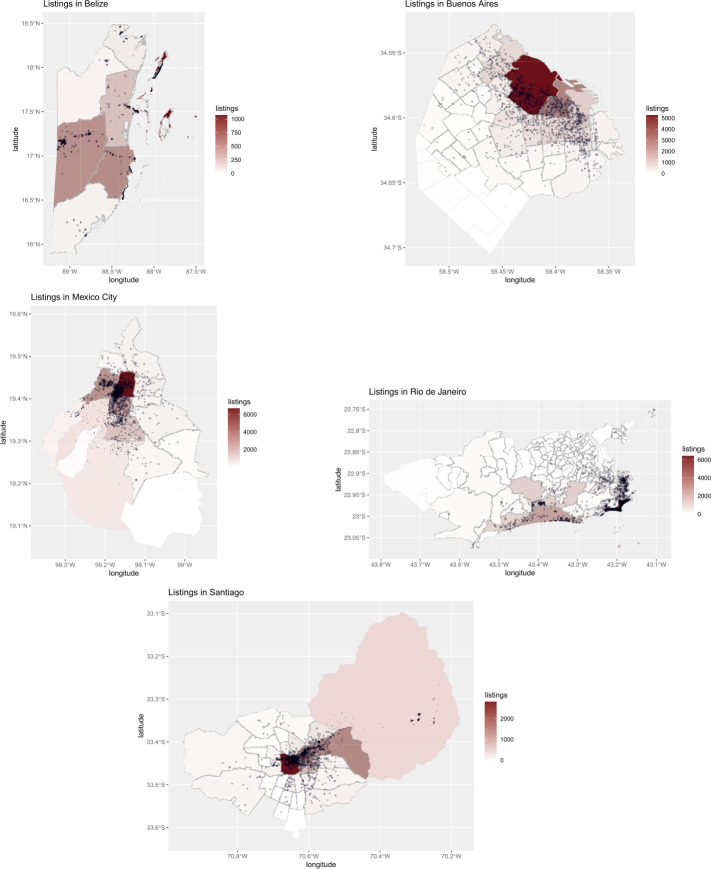
Offer (host) density in the five Latin American cities analysed

**Figure 16:**
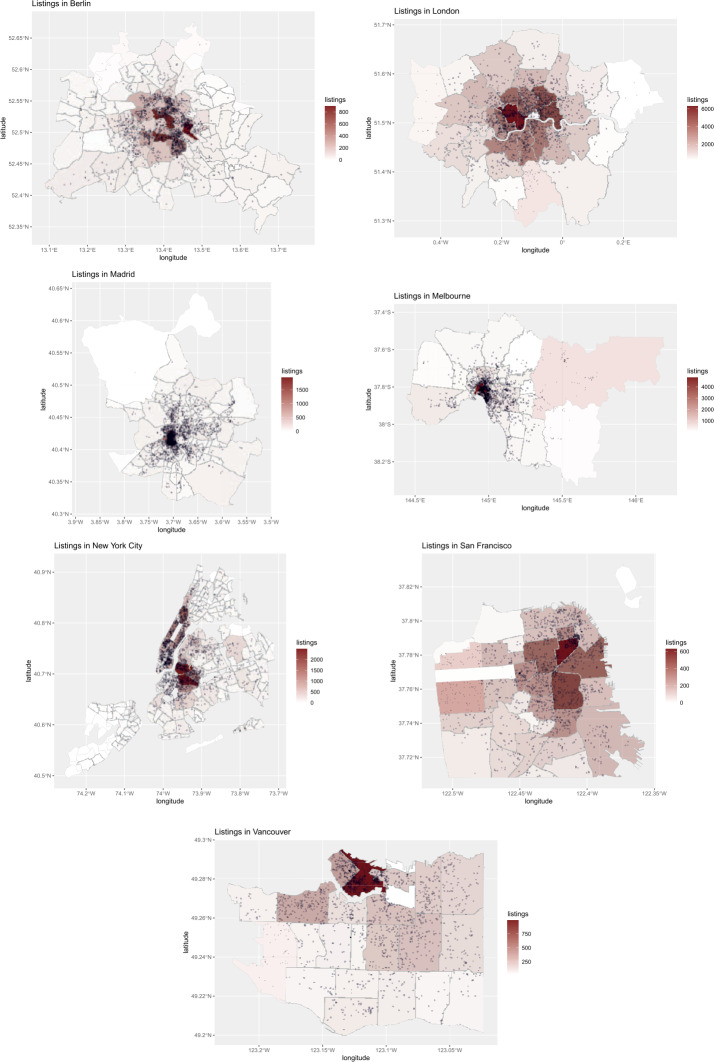
Offer (host) density in the seven Western cities analysed

**Figure 17:**
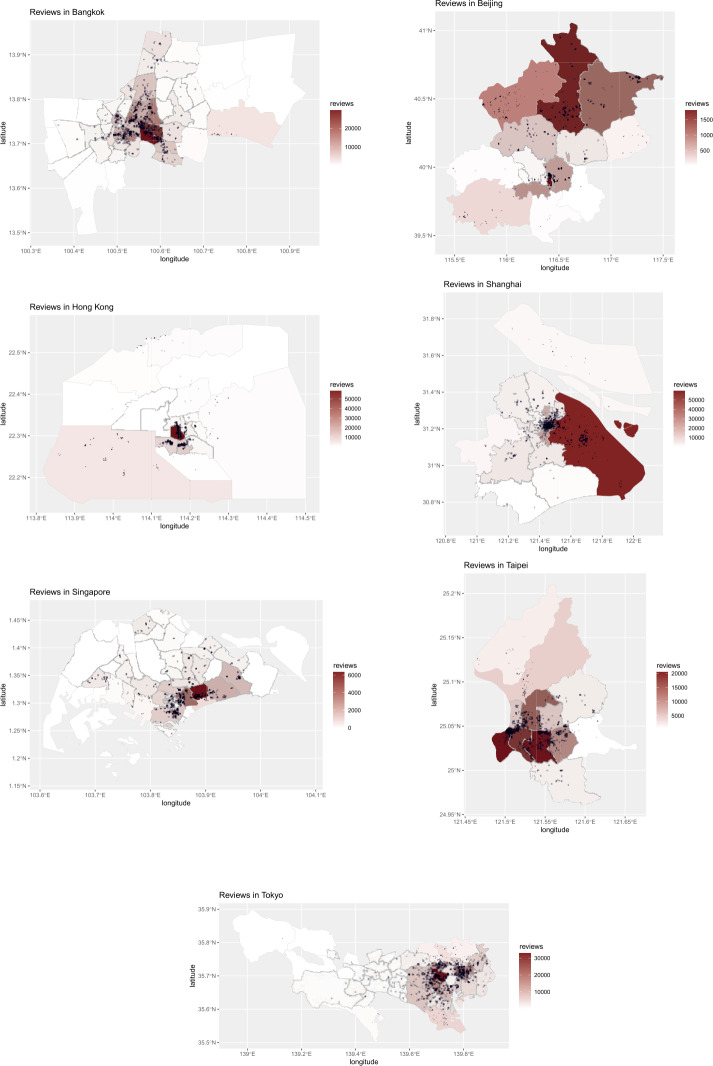
Demand (guest) density in the seven Asian cities analysed

**Figure 18:**
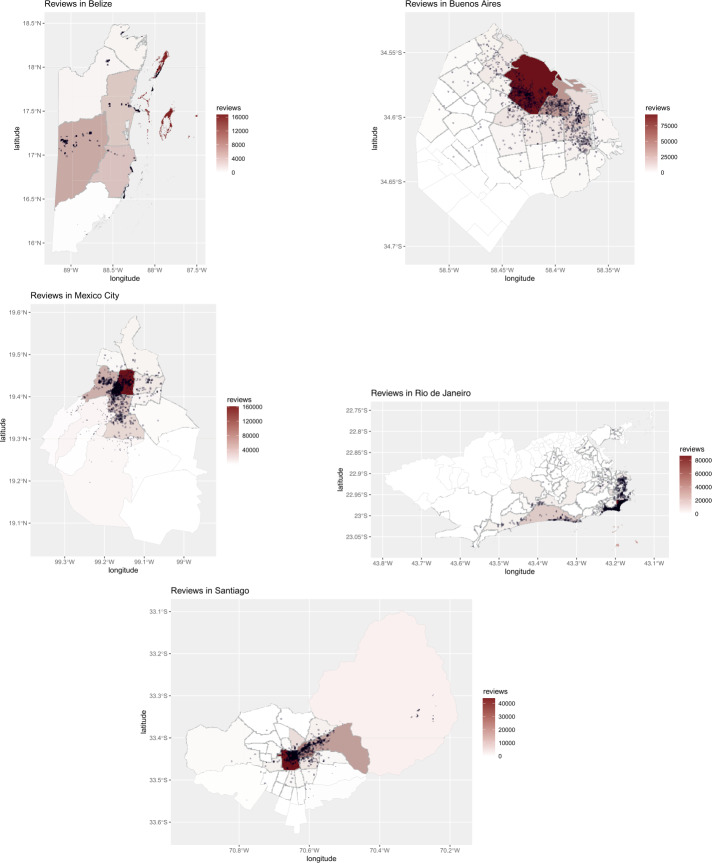
Demand (guest) density in the five Latin American cities analysed

**Figure 19:**
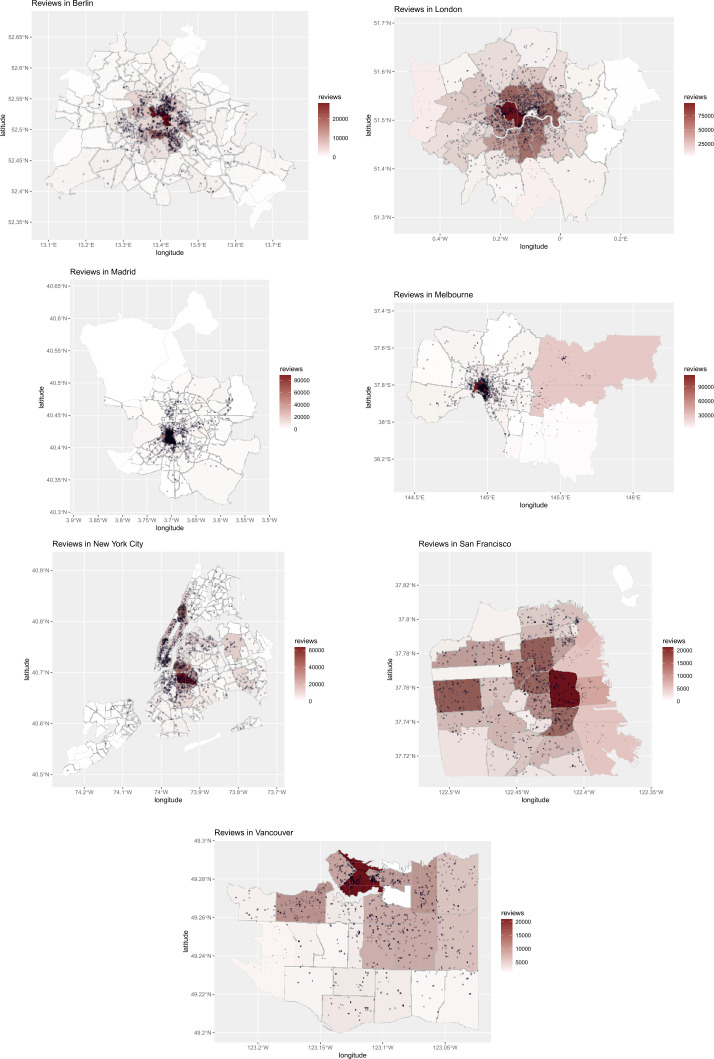
Demand (guest) density in the seven Western cities analysed

## Data Availability

The code used to scrape Airbnb data will be made available in a public repository after acceptance.

## Appendix

Figs. 14–19 show, as supplementary material, the offer and demand maps of all 19 cities under study.

## Footnotes

1 https://www.thezebra.com/resources/home/airbnb-statistics/

2 https://www.airbnb.co.uk/help/article/420/what-are-neighbourhoods

3 Maps of offer and demand for all 19 cities under study are available in the Appendix.

## Abbreviations

SE: Sharing Economy
UK: United Kingdom
US: United States of America
RQ1: Research Question 1
RQ2: Research Question 2
NLP: Natural Language Processing
GSDMM: Gibbs Sampling DirichletMixture Model
LDA: Latent Dirichlet Allocation
